# A Mendelian randomization study of the effect of tea intake on breast cancer

**DOI:** 10.3389/fnut.2022.956969

**Published:** 2022-10-18

**Authors:** Yuqing Deng, Wenxin Ge, Huili Xu, Jiaming Zhang

**Affiliations:** ^1^Department of Thyroid Breast Surgery, The Central Hospital of Wuhan, Tongji Medical College, Huazhong University of Science and Technology, Wuhan, China; ^2^Department of Urology, The Central Hospital of Wuhan, Tongji Medical College, Huazhong University of Science and Technology, Wuhan, China; ^3^Department of Surgery, The Central Hospital of Wuhan, Tongji Medical College, Huazhong University of Science and Technology, Wuhan, China

**Keywords:** tea consumption, breast cancer, single nucleotide polymorphisms, Mendelian randomization, causal association

## Abstract

**Background:**

The relationship between tea consumption and the risk of breast cancer is inconsistent in previous observational studies and is still in dispute. We intended to detect the causal association between tea consumption and breast cancer risk using two-sample Mendelian randomization (MR) analysis.

**Materials and methods:**

The summary statistics of tea consumption was obtained from the UK Biobank Consortium with 349,376 individuals and breast cancer information was obtained from the Breast Cancer Association Consortium (BCAC) (122,977 cases and 105,974 non-cases). Sensitivity analyses of evaluating the influence of outliers and pleiotropy effects were performed by a variety of MR methods under different model assumptions.

**Results:**

After potentially excluding pleiotropic single nucleotide polymorphisms (SNPs) using the MR Pleiotropy RESidual Sum and Outlier method, the odds ratio (OR) for per extra daily cup of tea intake for overall, estrogen receptor (ER)-positive, and ER-negative breast cancer risk was 1.029 [95% confidence interval (CI) = 0.983–1.077, *P* = 0.2086], 1.050 (95% CI = 0.994–1.109, *P* = 0.078), and 1.081 (95% CI = 0.990–1.103, *P* = 0.6513), respectively. The results were consistent with a sensitivity analysis that excluded SNPs associated with other phenotypes, manifesting that the findings were convincing and robust. Moreover, in the multivariable MR analysis, the null associations for breast cancer risk remained after adjusting for smoking and alcohol consumption separately or together.

**Conclusion:**

Our MR results based on genetic data did not support a causal relationship between tea consumption and breast cancer risk.

## Introduction

Breast cancer is the most common malignant tumor in women and the second leading cause of death worldwide (1), with an estimated incidence of 2,179,457 new cases and 655,690 deaths in 2020 (2, 3). The incidence of breast cancer is increasing in both developing and developed countries due to improved life expectancy, urbanization, and lifestyle changes (4).

Tea is one of the most widely consumed beverages around the world. Tea has been believed to have a variety of health benefits and has been applied to medical purposes (5). Several studies have shown that the compounds of tea have anti-cancer effects (6–8). However, there is an unclear consensus in the epidemiological studies on whether tea consumption is beneficial to population health, especially for cancer (8). Several meta-analyses showed that tea was not related to breast cancer risk (9–13), some studies reported an inverse effect against breast cancer (14–16), and several studies showed a positive association (17). The reliability of these findings can be easily influenced by cross-sectional design, small sample size, insufficient follow-up period, or different reference groups. In addition, given the limitations of observational studies and the potential effects of reverse causality or confounding factors, conclusions cannot be drawn for a causal association between tea consumption and breast cancer risk.

Mendelian randomization (MR) design employs genetic variants as instrumental variables (IVs) for exposure and can enhance causal inference. Residual confounding is minimized, and MR is less affected by reverse causation because genetic variants are randomly assigned at conception, so that a trait is generally not related to other traits (potential confounding factors or environmental factors). Therefore, we performed an MR study to assess the association of tea intake with breast cancer risk.

## Materials and methods

### Genetic instrument selection

**FIGURE 1:**
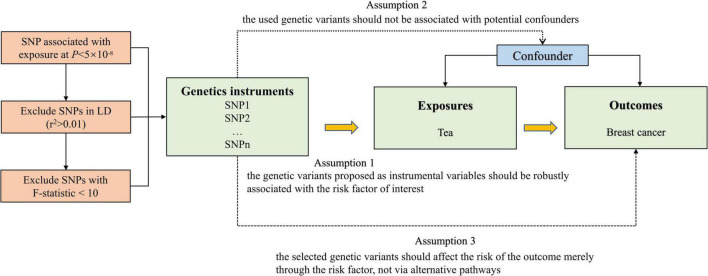
Overview and assumptions of the Mendelian randomization study design.

The flow chart of our MR study design is displayed in Figure 1. The genome-wide association study (GWAS) summary-level data set for tea consumption was obtained from UK Biobank Consortium (phenotype Code: 1488_raw). The data set was obtained from Neale Lab (GWAS round 2), and the participants were over 349,376 individuals of European ancestry.^1^ We obtained the data of habitual tea consumption from a dietary questionnaire. The questionnaire included a question: “How many cups of tea (including black and green tea) do you drink per day?” Summary statistics of daily unconverted tea consumption by GWAS were employed to identify single nucleotide polymorphisms (SNPs) associated with tea drinking. The GWAS was adjusted for age, sex, and the first 20 ancestral principal components. We chose autosomal biallelic SNPs with *P* < 5 × 10^–8^ and further conducted quality control at a minor frequency >1%. In addition, based on the reference data from the European sample in the 1000 Genomes Project, we clumped these SNPs with linkage disequilibrium *r*^2^ < 0.01 at a 1,000 kb window. At last, 45 independent SNPs strongly related to tea consumption were used in our primary analysis (Supplementary Table 1).

### Genetic summary-level data of breast cancer

We acquired the largest publicly available dataset on breast cancer from the Breast Cancer Association Consortium (BCAC), and no additional ethics approval and informed consent were required (18). The consortium provided summary-level association statistics for overall (122,977 cases and 105,974 non-cases), estrogen receptor (ER)-positive (69,501 breast cancer cases), and ER-negative breast cancer population (21,468 breast cancer cases) (15). The study included the combined results of OncoArray (61,282 cases and 45,494 non-cases) and the iCOGS dataset (46,785 cases and 42,892 non-cases), as well as 11 additional GWASs (14,910 cases and 17,588 non-cases). To minimize ancestry mismatches, the current analysis was limited to female subjects of European descent.

### Other factors

When the previous MR studies showed significant associations with common causes of tea consumption and breast cancer risk, we performed multivariable MR analysis to adjust for indirect pathways, which were likely to introduce correlated pleiotropy (19). The previous MR studies revealed relationships of instruments with smoking and alcohol drinking-related traits (20–22). Therefore, we selected SNPs for smoking per day from a GWAS of 337,334 individuals and SNPs for drinks per week from a GWAS of 941,280 individuals for the multivariable MR analyses (23). Finally, 21 and 39 SNPs were identified as IVs for smoking and alcohol consumption at a genome-wide significance level with a *P*-value < 5 × 10^–8^, following clumping for LD at *r*^2^ < 0.01.

### Statistical analyses

We calculated the variance in tea consumption explained by the IVs, and used the F-statistics to test weak IVs bias. *F*-value > 10 was suggested to be a strong genetic IV (24), and we deleted SNPs with an *F*-value less than 10 to fulfill the assumption of the first assumption of MR. We conducted a power analysis of the MR to identify a non-zero causal association of tea consumption with breast cancer by an online tool^2^ (25). We applied MR-Steiger analysis to monitor the direction of the potential causal effect between tea consumption and breast cancer risk. Cochran’s *Q*-test was used to quantify the size of heterogeneity between the genetic IVs. Random-effect inverse-variance-weighted (IVW) model was used as the main analytical method to examine the causal association. The random-effect IVW method included individual MR effects of SNPs to derive overall weighted effects. Potential violation of the second and third assumptions of MR was examined using several approaches, such as the weighted median, simple median, and the MR-Egger regression methods (26–29). When the Egger-intercept of the linear regression was close to 0, there was no directional pleiotropy of the IVs, and the exclusivity hypothesis can be considered to be valid (29). We used two steps for sensitivity analysis. First, MR Pleiotropy RESidual Sum and Outlier (MR-PRESSO) analysis was used for outlier detection and to generate inverse-variance weighted estimates after the removal of outliers (30). We also used the *P*-value from MR-PRESSO distortion test to test whether there was a significant difference between the estimates before and after outlier correction. We removed potential outliers and performed the first sensitivity analysis among the rest of the SNPs. Second, in order to verify whether the IVs satisfied the independence assumption, we searched PhenoSacnner^3^ web (Supplementary Table 2). SNPs related to traits other than tea consumption were recorded at the significance level (*P* < 5 × 10^–8^). We removed these SNPs and performed the second sensitivity analysis. Multivariable MR can then be used to adjust for pleiotropy (31, 32). We used multivariable IVW analyses to adjust for the genetic correlation between the smoking status and alcohol consumption. Odds ratios (ORs) were scaled per cup increment in daily tea consumption. Bonferroni correction (*P* = 0.05/3 outcomes) was used to adjust for multiple testing (*P* = 0.017) in this MR. All statistical tests were two-sided and performed using the TwoSampleMR and MR-PRESSO packages in R Software 3.6.1.

## Results

### Selection of instrumental variables

The *F*-values of the obtained SNPs were all greater than 10, suggesting that there could be no bias caused by weak IVs (Supplementary Table 1).

### Steiger-Mendelian randomization analysis

The Steiger-MR analysis was applied to detect the robustness of the causal effect estimates. Steiger-MR identified that the SNPs explained more variance in exposure than outcome (all *P* > 0.05).

### Power analyses

Using the mRnd method, we calculated the phenotypic variance explained to 0.76%, which was equal to the total phenotypic variance of tea consumption explained by all the valid IVs. The OR was 1.14, 1.16, and 1.28 for overall, ER-positive, and ER-negative breast cancer, respectively, when the estimated statistical power was 80% with the current sample size.

### Causal effect in the main analysis

**FIGURE 2:**
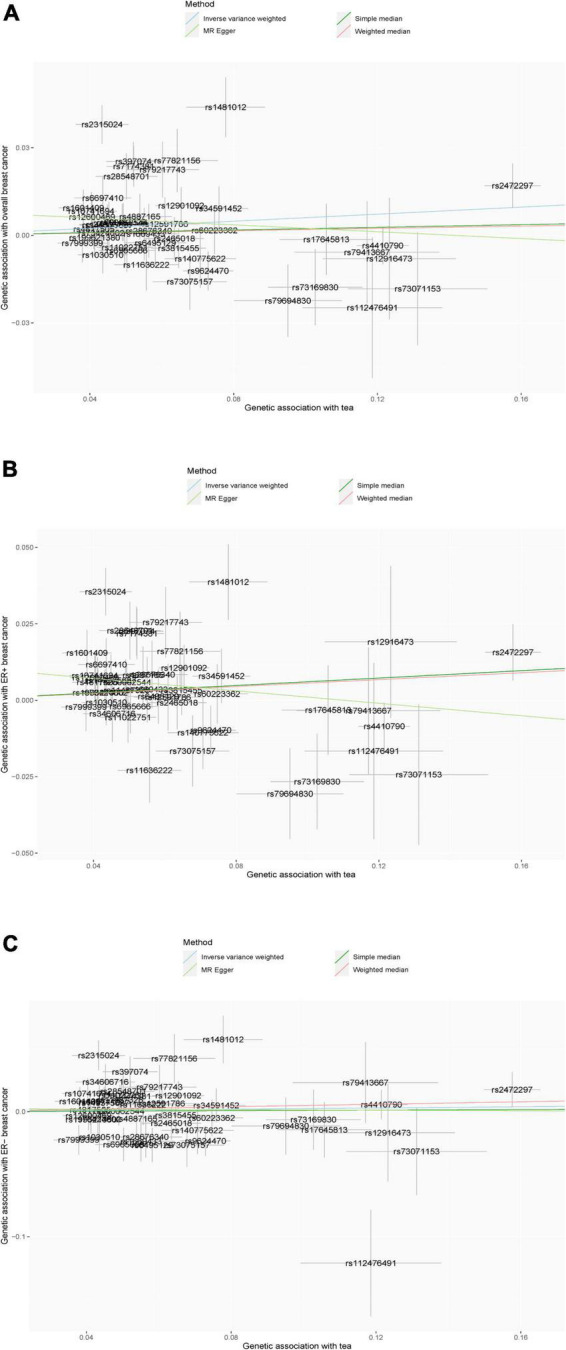
**(A–C)** Scatter plot of the results of Mendelian randomization analysis. **(A)** Overall breast cancer; **(B)** ER-positive breast cancer; and **(C)** ER-negative breast cancer.

**FIGURE 3:**
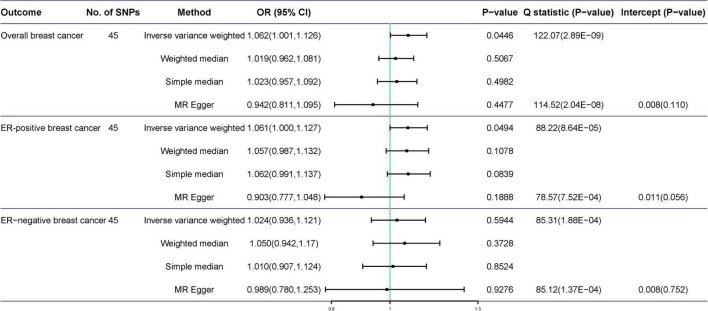
Mendelian randomization of tea consumption and breast cancer in the primary analysis.

The scatter plot of the SNP-breast cancer association against the SNP-tea association is shown in Figure 2. The estimates of the causal effect of genetically predicted tea consumption on breast cancer risk are shown in Figure 3. Significant heterogeneity was detected between tea consumption and overall, ER-positive, and ER-negative breast cancer by the Cochran heterogeneity test through IVW and MR-Egger method (Figure 3). The MR-Egger method showed no significant horizontal pleiotropy in the association between tea consumption and breast cancer (Figure 3). Weakly increased risk for overall [OR_*IVW*_ = 1.062, 95% confidence interval (CI) = 1.001–1.126, *P* = 0.0446] and ER-positive breast cancer (OR_*IVW*_ = 1.061, 95% CI = 1.000–1.127, *P* = 0.0494) was observed, but there was no association between tea consumption and ER-negative breast cancer risk (OR_*IVW*_ = 1.024, 95% CI = 0.936–1.121, *P* = 0.065) (Figure 3). Using the weighted median, simple median, and MR-Egger regression methods, we found no association between tea consumption and overall, ER-positive, and ER-negative breast cancer risk.

### Causal effect in the sensitivity analyses

**FIGURE 4:**
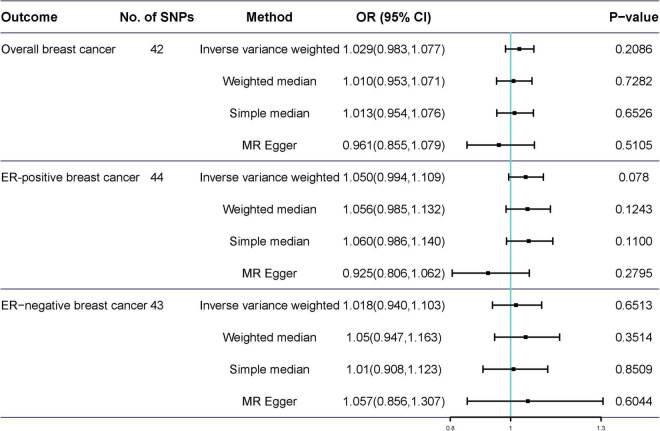
Mendelian randomization of tea consumption and breast cancer in the sensitivity analysis. Three, one, and two outliers were detected in the MR-PRESSO analysis of tea consumption and overall, ER-positive, and ER-negative breast cancer.

**FIGURE 5:**
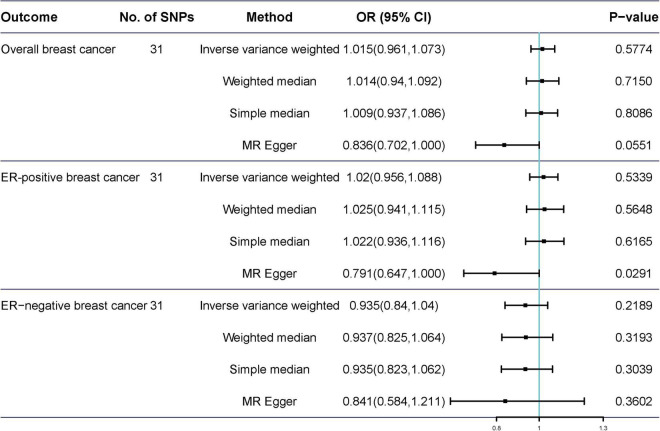
Mendelian randomization results of tea consumption and breast cancer after excluding SNP with potential pleiotropy.

Potentially pleiotropic SNPs were excluded using MR-PRESSO. Specifically, rs199621380, rs2315024, and rs397074 were excluded from the analysis of overall breast cancer; rs2315024 was excluded from the analysis of ER-positive breast cancer; and rs112476491 and rs2315024 were excluded from the analysis of ER-negative breast cancer. We found no differences in estimates before and after the removal of outliers in these analyses (*P* for MR-PRESSO distortion tests >0.05). The OR of overall, ER-positive, and ER-negative breast cancer was 1.030 (0.984–1.078, *P* = 0.2158), 1.050 (0.995–1.109, *P* = 0.0852), and 1.025 (0.361–2.907, *P* = 0.597) in the outlier-corrected MR-PRESSO analysis, respectively. Using the existing SNPs as IVs, the results indicated that tea consumption was not associated with overall (OR_*IVW*_ = 1.029, 95% CI = 0.983–1.077, *P* = 0.2086), ER-positive (OR_*IVW*_ = 1.050, 95% CI = 0.994–1.109, *P* = 0.078), and ER-negative breast cancer risk (OR_*IVW*_ = 1.081, 95% CI = 0.990–1.103, *P* = 0.6513) (Figure 4). In addition, using the weighted median, simple median, and MR-Egger regression methods, we found there was no correlation between tea consumption and overall, ER-positive, and ER-negative breast cancer. Moreover, after excluding SNPs associated with other phenotypes, the results did not show significant changes (Figure 5).

### Multivariable Mendelian randomization analysis

We performed multivariable IVW analyses by adjusting for tobacco smoking and alcohol drinking separately or together. We did not observe statistically significant associations between genetic liability to tea consumption and breast cancer risk in any of these analyses (Table 1).

**TABLE 1:** Effect estimates for the association between genetic predisposition to tea consumption and the risk of breast cancer in the multivariable MR analysis.

Outcome	Model	OR (95% CI)	*P*-value
Overall breast cancer	IVW adjusted for smoking	1.164 (0.981, 1.381)	0.081
	IVW adjusted for alcohol drinking	0.985 (0.817, 1.186)	0.869
	IVW fully adjusted model	1.175 (0.983, 1.378)	0.472
ER-positive breast cancer	IVW adjusted for smoking	1.164 (0.983, 1.365)	0.078
	IVW adjusted for alcohol drinking	1.205 (0.982, 1.437)	0.074
	IVW fully adjusted model	1.178 (1.001, 1.387)	0.085
ER-negative breast cancer	IVW adjusted for smoking	1.086 (0.830, 1.420)	0.548
	IVW adjusted for alcohol drinking	1.113 (0.866, 1.431)	0.404
	IVW fully adjusted model	1.087 (0.858, 1.377)	0.491

IVW, inverse-variance weighted; OR, odds ratio.

## Discussion

In this study, we applied a two-sample MR analysis using summary-level data from BCAC Consortium to evaluate the causal relationship of tea consumption with overall, ER-positive, and ER-negative breast cancer. The MR analysis showed that an extra daily cup of tea consumption seemed to be not protective against breast cancer.

Our research findings were consistent with several previous epidemiological studies, which found a null relationship between tea intake and breast cancer. A recent meta-analysis included one clinical trial and four cohort studies (10). In one clinical trial, green tea treatment did not alter mammogram density compared with placebo. In the four cohort studies, there was no significant difference in the risk of breast cancer among women who drank the highest level of green tea compared to those who drank the lowest level. In addition, a meta-analysis that combined the results of nine observational studies showed no significant relationship between green tea consumption and the risk of breast cancer (13). Another meta-analysis showed that drinking black tea did not reduce breast cancer risk in the United States and Europe (11). Additionally, the summary results of all cohort studies or case-control studies showed no association of black tea intake with breast cancer risk (11). A network meta-analysis that included 45 cohort and case-control studies found that tea drinking was not related to a lower overall breast cancer risk in postmenopausal women (9).

However, the relationship between tea consumption and the risk of breast cancer remains inconsistent in previous meta-analyses (14–16). Three meta-analyses found that individuals with the habit of drinking green tea were negatively associated with future breast cancer risk (14–16). In these three meta-analyses, most studies were conducted in Japan, China, and Singapore. Our MR analysis was conducted on data entirely derived from the European populations. This suggests two distinctions that may explain some of the discordances of prior results in the literature. Breast cancer, its presentation, and detection may be different in Asian populations compared to that in European populations. In addition, Asian populations mainly consume varieties of green tea, whereas European populations mainly consume black tea, and the constituents of these teas and their modes of consumption may have different effects. There may also be many confounding effects, such as Europeans consuming black tea with added milk, whereas Asians consume tea without milk products. Apart from that, most of these observational studies relied on self-report tea consumption assessment methods, which were prone to measurement error. Moreover, causality cannot be ascertained from such observational analyses, as they are vulnerable to residual confounding and reverse causality. The relationship between various components of tea and breast cancer risk requires further investigation.

Multiple potential biological mechanisms may explain the inverse relationship between tea consumption and breast cancer risk. Polyphenols or tea catechins are the main antioxidants in tea, which act as reactive oxygen species scavengers and may affect transcription factors and enzymatic activities (33). Epigallocatechin gallate is the most abundant catechin in tea and is believed to play a key role in inhibiting the occurrence and development of cancer (34, 35). Tea polyphenols are believed to inhibit the growth of tumor cells through a variety of mechanisms, such as inhibition of receptor-dependent signaling pathways and angiogenesis (36), induction of tumor cell apoptosis (37), silencing of genes related to epigenetic mechanisms, such as methylating DNA (38), and inhibiting enzyme activity (39). However, more mechanistic studies are needed.

This study has several advantages. First, this is the first attempt to explore the causal association between tea consumption and breast cancer through a two-sample MR analysis using GWAS summary-level statistics. Two-sample method, with large summary-level genetic data, is able to minimize potential confounders and reverse causality. Second, the findings were verified through various sensitivity analyses that applied multiple MR methods with different model assumptions, and the effects of outliers and pleiotropy were evaluated comprehensively. All the analyses indicated that the findings were consistent and robust.

However, this study had some limitations. First, we emphasize that our analytical power was relatively limited in observing the weak effect of tea consumption on breast cancer, which might be due in part to the lower proportion of tea consumption variability explained by valid SNPs. Second, it is important to note that our study was mainly based on the available GWAS data. There are no GWASs for different types of tea, making it difficult to infer the differential effects of tea type on the causal relationship between tea consumption and breast cancer. Third, the results cannot be generalized to other populations due to biases with limited data from the European population. Fourth, we could not conduct analyses of non-linear association or stratification effects due to the use of summary-level data. Fourth, we did not find sex-specific genetic IVs for tea consumption from the currently publicly available GWAS, which only included female subjects. Therefore, sex-combined IVs might lead to biased causal effect estimates in sex-specific two-sample MR studies. Future GWAS studies on tea consumption need to distinguish between male and female subjects. Finally, the MR results reflect the effect of lifelong tea intake on breast cancer. Therefore, the short-term effect of tea consumption on breast cancer requires further research.

## Conclusion

In conclusion, this MR study provides genetic evidence for a null causal relationship between tea consumption and breast cancer. Our results suggest that the evidence for tea consumption as a preventive measure for breast cancer is still deficient. Previous findings on the relationship between tea consumption and breast cancer risk may be influenced by confounding factors. More experimental studies are needed to confirm our findings.

## Data availability statement

The original contributions presented in this study are included in the article/Supplementary material, further inquiries can be directed to the corresponding authors.

## Ethics statement

Our study only adopts publicly available data. Ethical approval could be found in the original publications. Informed consent was obtained from all subjects involved in the study.

## Author contributions

JZ and HX conceived and designed the study and interpreted the results of the data analyses. YD performed the data analyses. YD and WG wrote the manuscript. All authors read and approved the final version of the manuscript.

## Footnotes

1
http://www.nealelab.is/uk-biobank

2
http://cnsgenomics.com/shiny/mRnd/

3
http://www.phenoscanner.medschl.cam.ac.uk
