# Supplementary material for: A Mendelian randomization study of the effect of tea intake on breast cancer
Source: Front Nutr. 2022 Oct 18;9:956969. doi: 10.3389/fnut.2022.956969 (PMC9623097; doi:10.3389/fnut.2022.956969)
Supplement: Supplementary file 1 [file Data_Sheet_1.docx]

Supplementary Material

# Supplementary Tables

**Supplementary Table 1.** Summary statistics for the single-nucleotide polymorphisms associated with tea consumption in the present Mendelian randomization study

| **SNP** | **EAF** | **EA** | **OA** | **BETA** | **SE** | **P** | **chr** | **Position** | **N** | **R^2^** | **F-statistic** |
| --- | --- | --- | --- | --- | --- | --- | --- | --- | --- | --- | --- |
| rs1030510 | 0.45 | G | A | -0.0436 | 0.0069 | 3.60E-10 | 7 | 17100273 | 349376 | 0.000114 | 40 |
| rs112476491 | 0.03 | A | G | -0.1186 | 0.0194 | 8.88E-10 | 7 | 17204040 | 349376 | 0.000107 | 37 |
| rs12591786 | 0.16 | T | C | -0.0609 | 0.0096 | 2.32E-10 | 15 | 60902512 | 349376 | 0.000115 | 40 |
| rs12916473 | 0.04 | A | G | 0.1233 | 0.0185 | 2.63E-11 | 15 | 75321999 | 349376 | 0.000127 | 44 |
| rs17645813 | 0.08 | A | G | -0.1058 | 0.013 | 3.32E-16 | 7 | 17419697 | 349376 | 0.00019 | 66 |
| rs73071153 | 0.03 | A | G | -0.1312 | 0.0194 | 1.32E-11 | 7 | 17545964 | 349376 | 0.000131 | 46 |
| rs2472297 | 0.27 | T | C | 0.1576 | 0.0078 | 3.82E-91 | 15 | 75027880 | 349376 | 0.00117 | 408 |
| rs28676340 | 0.16 | G | A | -0.0564 | 0.01 | 1.96E-08 | 15 | 75449794 | 349376 | 0.000091 | 32 |
| rs73424602 | 0.4 | T | C | -0.0432 | 0.007 | 7.84E-10 | 22 | 41461176 | 349376 | 0.000109 | 38 |
| rs149375687 | 0.27 | T | G | -0.0449 | 0.0078 | 7.26E-09 | 5 | 152034989 | 349376 | 9.48E-05 | 33 |
| rs200062544 | 0.47 | A | G | 0.049 | 0.007 | 2.64E-12 | 7 | 17260246 | 349376 | 0.00014 | 49 |
| rs9624470 | 0.58 | A | G | 0.0729 | 0.007 | 3.06E-25 | 22 | 24820268 | 349376 | 0.00031 | 108 |
| rs10741694 | 0.63 | C | T | 0.0404 | 0.0071 | 1.53E-08 | 11 | 16286183 | 349376 | 9.27E-05 | 32 |
| rs11022751 | 0.27 | C | T | 0.0497 | 0.0078 | 1.83E-10 | 11 | 13307613 | 349376 | 0.000116 | 41 |
| rs79413667 | 0.03 | G | C | -0.1171 | 0.0201 | 6.03E-09 | 7 | 17399486 | 349376 | 9.71E-05 | 34 |
| rs11487328 | 0.38 | C | G | -0.0493 | 0.0071 | 5.16E-12 | 1 | 174601659 | 349376 | 0.000138 | 48 |
| rs11636222 | 0.23 | G | T | -0.0557 | 0.0089 | 3.79E-10 | 15 | 75515312 | 349376 | 0.000112 | 39 |
| rs3815455 | 0.29 | T | C | 0.0647 | 0.0076 | 1.74E-17 | 7 | 75611756 | 349376 | 0.000207 | 72 |
| rs12600469 | 0.62 | T | G | 0.0406 | 0.0071 | 1.22E-08 | 17 | 40834073 | 349376 | 9.36E-05 | 33 |
| rs12901092 | 0.39 | A | C | -0.0654 | 0.0071 | 3.20E-20 | 15 | 75374145 | 349376 | 0.000243 | 85 |
| rs6965666 | 0.28 | C | T | -0.0503 | 0.0078 | 9.14E-11 | 7 | 17177312 | 349376 | 0.000119 | 42 |
| rs140775622 | 0.17 | T | C | 0.0707 | 0.0099 | 9.33E-13 | 20 | 62962869 | 349376 | 0.000146 | 51 |
| rs1481012 | 0.11 | G | A | -0.0778 | 0.0109 | 9.41E-13 | 4 | 89039082 | 349376 | 0.000146 | 51 |
| rs1601409 | 0.46 | G | A | 0.0382 | 0.0069 | 3.67E-08 | 12 | 17066769 | 349376 | 8.77E-05 | 31 |
| rs1669433 | 0.84 | G | A | 0.0551 | 0.0093 | 3.33E-09 | 12 | 11349732 | 349376 | 0.0001 | 35 |
| rs4887165 | 0.81 | C | T | 0.0539 | 0.0089 | 1.22E-09 | 15 | 74889356 | 349376 | 0.000105 | 37 |
| rs199621380 | 0.41 | G | T | 0.0413 | 0.007 | 4.53E-09 | 1 | 150700614 | 349376 | 9.96E-05 | 35 |
| rs4410790 | 0.63 | C | T | 0.1215 | 0.0072 | 1.89E-64 | 7 | 17284577 | 349376 | 0.000814 | 285 |
| rs2315024 | 0.33 | A | T | 0.0434 | 0.0073 | 2.98E-09 | 19 | 19423817 | 349376 | 0.000101 | 35 |
| rs2465018 | 0.23 | A | G | 0.0635 | 0.0082 | 1.38E-14 | 6 | 51241140 | 349376 | 0.000172 | 60 |
| rs79217743 | 0.14 | T | G | -0.0602 | 0.0102 | 3.34E-09 | 15 | 75117912 | 349376 | 9.97E-05 | 35 |
| rs28548701 | 0.8 | C | T | -0.0502 | 0.0086 | 5.82E-09 | 15 | 74346021 | 349376 | 9.75E-05 | 34 |
| rs6495129 | 0.2 | T | G | -0.0582 | 0.0086 | 1.35E-11 | 15 | 75196717 | 349376 | 0.000131 | 46 |
| rs34591452 | 0.24 | T | G | 0.0759 | 0.0081 | 5.48E-21 | 15 | 74492585 | 349376 | 0.000251 | 88 |
| rs34606716 | 0.24 | A | G | -0.0453 | 0.0082 | 2.70E-08 | 7 | 75820449 | 349376 | 8.73E-05 | 31 |
| rs77821156 | 0.11 | G | A | 0.0643 | 0.0113 | 1.39E-08 | 7 | 17331450 | 349376 | 9.27E-05 | 32 |
| rs397074 | 0.31 | C | G | -0.0521 | 0.0075 | 2.80E-12 | 15 | 74599997 | 349376 | 0.000138 | 48 |
| rs7999399 | 0.55 | T | C | 0.0379 | 0.0069 | 4.96E-08 | 13 | 89233505 | 349376 | 8.63E-05 | 30 |
| rs73169830 | 0.08 | C | T | 0.1027 | 0.0131 | 3.81E-15 | 22 | 24885208 | 349376 | 0.000176 | 61 |
| rs4817505 | 0.39 | C | T | 0.0411 | 0.0071 | 6.22E-09 | 21 | 34343828 | 349376 | 9.59E-05 | 34 |
| rs79694830 | 0.06 | T | C | 0.0951 | 0.015 | 2.26E-10 | 7 | 17286087 | 349376 | 0.000115 | 40 |
| rs60223362 | 0.2 | C | T | -0.0747 | 0.0086 | 5.35E-18 | 7 | 17459648 | 349376 | 0.000216 | 75 |
| rs73075157 | 0.13 | A | G | -0.0678 | 0.0103 | 5.42E-11 | 7 | 17566844 | 349376 | 0.000124 | 43 |
| rs6697410 | 0.74 | T | G | 0.0436 | 0.0079 | 4.10E-08 | 1 | 26756209 | 349376 | 8.72E-05 | 30 |
| rs7174381 | 0.31 | C | A | 0.0522 | 0.0075 | 3.85E-12 | 15 | 75613289 | 349376 | 0.000139 | 48 |

SNP, single-nucleotide polymorphism; Chr, chromosome; EA, effect allele; EAF, effect allele frequency; SE, standard error

R^2^ was calculated as follows:

2*beta^2^*EAF*(1-EAF)/(2*beta^2^*EAF*(1-EAF)+se^2^*2*N*EAF(1-EAF)).

The F-statistic for each SNP was calculated as follows: F=(N−2)*R^2^/(1−R^2^).

**Supplementary Table 2.** Potential secondary phenotypes of the genetic variants used for tea consumption

| **SNP** | **Trait** |
| --- | --- |
| rs10741694 | Hypertension |
| rs79217743 | Granulocyte percentage of myeloid white cells；Diastolic blood pressure |
| rs2315024 | Self-reported high cholesterol |
| rs1481012 | Cholesterol ldl |
| rs199621380 | Neutrophil percentage of granulocyte；Neutrophil percentage of white cells；Granulocyte percentage of myeloid white cells |
| rs3815455 | Creatinine in urine |
| rs2472297 | Creatinine in urine; Platelet distribution width |
| rs11022751 | Body mass index；Diastolic blood pressure；Age at menarche |
| rs12600469 | Weight |
| rs397074 | Hypertension |
| rs73424602 | Serum urate; Uric acid |
| rs4410790 | Creatinine in urine |
| rs7174381 | Creatinine in urine |
| rs9624470 | Creatinine in urine |
